# Supplementary material for: Self-driving laboratories to autonomously navigate the protein fitness landscape
Source: Nat Chem Eng. Author manuscript; Available in PMC 2024 Mar 11. (PMC10926838; doi:10.1038/s44286-023-00002-4)
Supplement: Supplementary Information & Figs. [file NIHMS1959490-supplement-Supplementary_Information___Figs_.pdf]

nature chemical engineering

Article

https://doi.org/10.1038/s44286-023-00002-4

Self-driving laboratories to autonomously navigate the protein fitness landscape

In the format provided by the authors and unedited

Table of Contents

Figure S1: Batch BO method performance Page 2

Figure S2: EvaGreen assay to test successful gene assembly and PCR amplification Page 2

Figure S3: Enzyme reaction progress curves Page 3

Figure S4: The contribution of individual gene fragments to enzyme thermostability and probability of being active Page 3

Figure S5: Enzyme reaction kinetics Page 4

Detailed description of SAMPLE code functionality Page 5

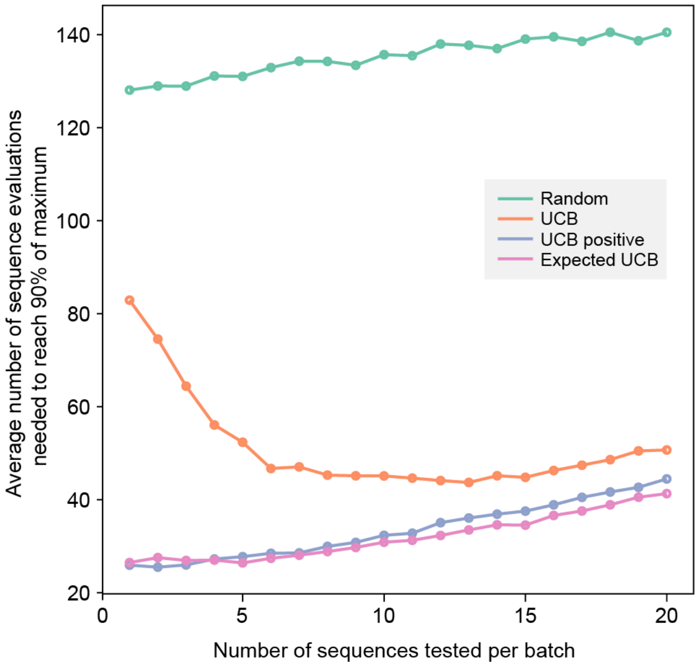

Figure S1: Batch BO method performance. We tested batch Bayesian Optimization (BO) methods by running 10,000 simulated protein engineering experiments with the cytochrome P450 data and evaluating how many sequence evaluations were needed to discover sequences within 90% of the maximum sequence (>61.9 °C). The Upper Confidence Bound (UCB) positive and Expected UCB methods show the best overall performance and perform better with smaller batch sizes.

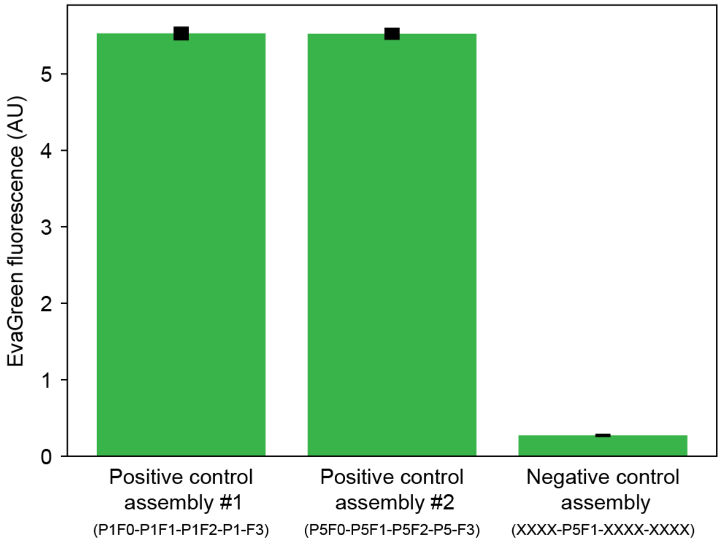

Figure S2: EvaGreen assay to test successful gene assembly and PCR amplification. We tested two positive control assemblies with DNA fragments to assemble full genes and a negative control assembly that only had one DNA fragment. The EvaGreen fluorescence clearly distinguishes between successful and unsuccessful gene assembly. Data are presented as mean ± 1 standard deviation of three measurements.

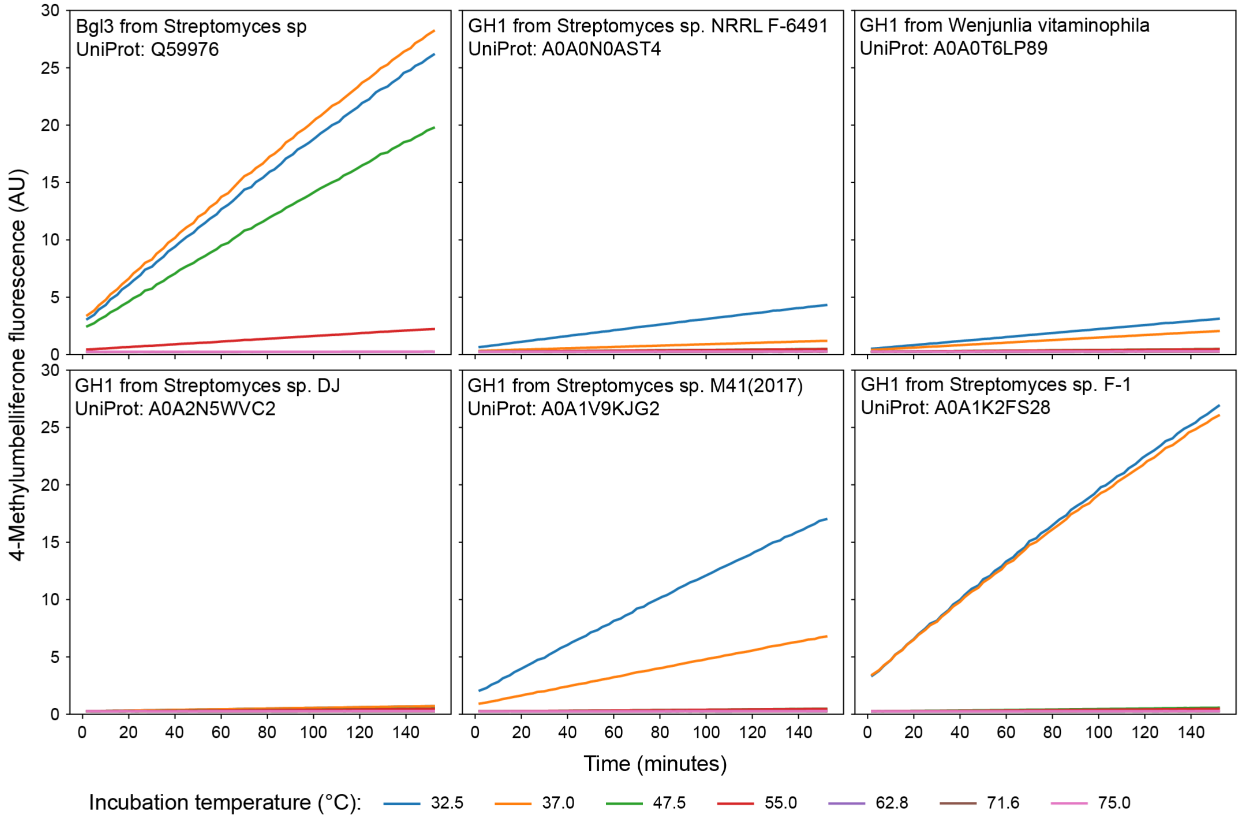

Figure S3: Enzyme reaction progress curves. An example of enzyme reaction progress curves for the six natural GH1s used to initialize the Bayesian optimization runs. Enzyme reactions were run at room temperature after a 10-minute incubation at the specified temperature. The decrease in activity is the result of irreversible enzyme inactivation from the temperature incubation.

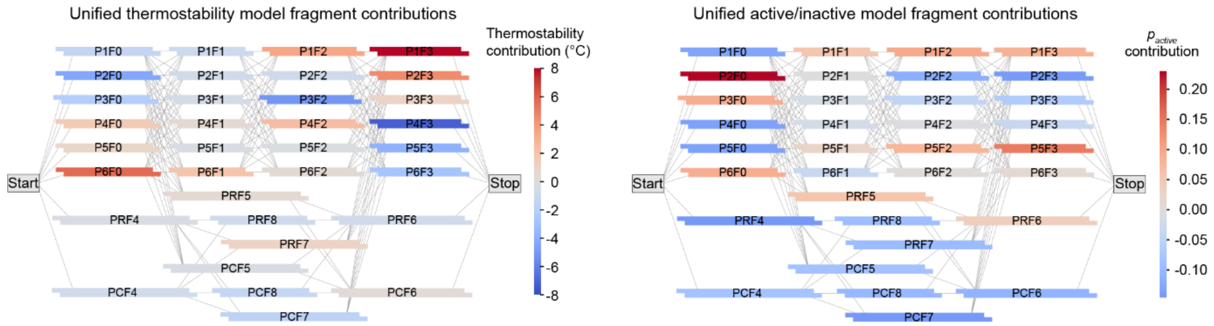

Figure S4: The contribution of individual gene fragments to enzyme thermostability and probability of being active. The unified landscape model was trained on all collected data across all agents and all runs. The fragment contributions are calculated as the mean of the property (thermostability or p_{active}) across all sequences subtracted from the mean over sequences that have that specific fragment.

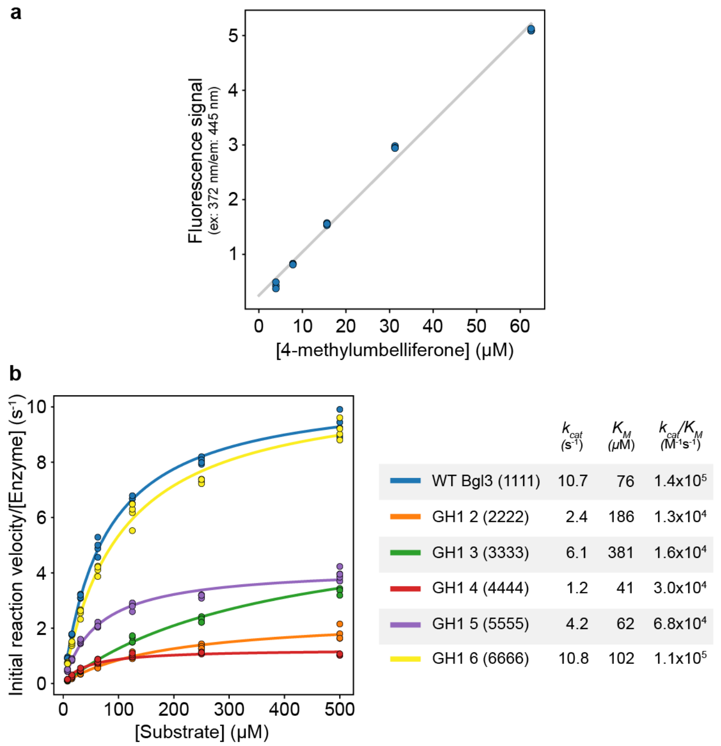

Figure S5: Enzyme reaction kinetics. (a) Standard curve of the 4-Methylumbelliferone fluorescent reaction product showing a linear relationship up to 60 μM. **(b) E**nzyme kinetics for the six wild-type input sequences. Each measurement was performed in triplicate and the Michaelis-Menten equation was fit to the average over replicates to determine the kinetic constants. Wild-type Bgl3 has the greatest catalytic efficiency (k_{cat}/K_m) and was used as a reference point in Fig 5b.

Detailed description of SAMPLE code's functionality

A pseudocode description of a single SAMPLE cycle is described below.

1. For each filename included as an argument (one for each agent):
   - Read all data the agent has previously collected and categorize all possible sequences into the three distinct sets:
     - Unexplored sequences
     - Active observed sequences with T50 values
     - Inactive observed sequences with no T50 values
   - To select a batch of sequences for experimental testing, repeat the following procedure until the desired number of sequences have been selected (batch size = 3 for this paper). If not working in batches, perform these steps only once.
     - Train a Gaussian process regressor (GPR) using only active observed sequences to predict T50 with uncertainty.
     - Train a Gaussian process classifier (GPC) using active and inactive observed sequences to predict the probability of being active.
     - Use both the GPR and GPC to predict the T50, uncertainty of T50, and probability of being active for all unobserved sequences.
     - Subtract the minimum predicted T50 from all T50 predictions (this is to ensure subsequent multiplication captures the full range of predictions)
     - For each sequence, calculate the eUCB score according to the formula ((u + 2d) * p) where u is the T50, d is the standard deviation of that prediction, and p is the probability of being active.
     - Select the sequence with the greatest eUCB for testing.
     - If not done with the current batch, assume the T50 prediction for the chosen sequence is correct, update the model accordingly, and begin the sequence selection cycle again to identify the next sequence in the batch.
2. Concatenate the list of each agent's chosen sequences together to get one list to submit for testing.
3. Record the chosen sequence list and submit a run to Strateos.
4. Check Strateos's list of runs every 60 seconds until five unique experiments (Golden gate assembly, PCR, EvaGreen assay, cell-free expression, and the enzyme thermostability assay) have been added to the queue.
5. Read the Strateos output from the third (EvaGreen assay) of those five experiments to determine which sequences were successfully assembled. This is the EvaGreen checkpoint step.
6. For those sequences that successfully assembled:
   - Fit raw slopes with fluorescence vs. time, one for each temperature tested.
   - Normalize raw slopes based on the fluorescein internal standard.
   - Fit a double logistic curve, plotting normalized slope vs. heating temperature. If fitting fails, label the sequence as retry and do not continue processing it.

   - If the fitted curve is too small, label the sequence retry if observed for the first time or dead if already labeled retry.
   - If all filters have been passed, take the T50 from the curve fit and label the sequence with its T50
7. Save all data from the run and begin the next. Repeat in this way until the desired number of cycles is completed.
